# Can Lessons from Public Health Disease Surveillance Be Applied to Environmental Public Health Tracking?

**DOI:** 10.1289/ehp.7450

**Published:** 2004-12-02

**Authors:** Beate Ritz, Ira Tager, John Balmes

**Affiliations:** ^1^Department of Epidemiology, University of California, Los Angeles, California, USA; ^2^Division of Epidemiology, and; ^3^Division of Environmental Health Sciences, School of Public Health, University of California, Berkeley, California, USA

**Keywords:** environmental health, evaluation, intervention, registries, surveillance

## Abstract

Disease surveillance has a century-long tradition in public health, and environmental data have been collected at a national level by the U.S. Environmental Protection Agency for several decades. Recently, the Centers for Disease Control and Prevention announced an initiative to develop a national environmental public health tracking (EPHT) network with “linkage” of existing environmental and chronic disease data as a central goal. On the basis of experience with long-established disease surveillance systems, in this article we suggest how a system capable of linking routinely collected disease and exposure data should be developed, but caution that formal linkage of data is not the only approach required for an effective EPHT program. The primary operational goal of EPHT has to be the “treatment” of the environment to prevent and/or reduce exposures and minimize population risk for developing chronic diseases. Chronic, multifactorial diseases do not lend themselves to data-driven evaluations of intervention strategies, time trends, exposure patterns, or identification of at-risk populations based only on routinely collected surveillance data. Thus, EPHT should be synonymous with a dynamic process requiring regular system updates to *a*) incorporate new technologies to improve population-level exposure and disease assessment, *b*) allow public dissemination of new data that become available, *c*) allow the policy community to address new and emerging exposures and disease “threads,” and *d*) evaluate the effectiveness of EPHT over some appropriate time interval. It will be necessary to weigh the benefits of surveillance against its costs, but the major challenge will be to maintain support for this important new system.

The Centers for Disease Control and Prevention (CDC) describes its own mission as serving “as the national focus for developing and applying disease prevention and control, environmental health, and health promotion and education activities designed to improve the health of the people of the United States” (CDC 2005). Recently, the CDC, for the first time, funded state and larger metropolitan health departments and three academic centers to begin to develop a national environmental public health tracking (EPHT) network. The CDC vision for the EPHT program is to improve protection of communities from adverse health effects through the integration of public health and environmental information systems. To implement this vision, the goal is to develop a national tracking (i.e., surveillance) network that links chronic disease and environmental data sources.

Surveillance has a long tradition in public health for both the descriptive epidemiology of diseases and the provision of insights into disease causation and disease control. It can be taken as axiomatic that, ultimately, all surveillance systems aim at disease control. Generally, surveillance refers to the continuous, routine collection of data related to health or exposures of populations over the long term, and the associated analysis, interpretation, and dissemination of the results. Surveillance data collected by government agencies such as the CDC and the U.S. Environmental Protection Agency (EPA) provide important archives that permit continued reinterpretation and health research. To date, however, the data systems established and used for surveillance focus either on diseases/syndromes or on media (e.g., ambient air pollutants, toxic agents) without formal linkage between systems. In this article, we focus our analyses mainly on properties and lessons learned from disease surveillance systems. We also provide arguments that effective surveillance does not always require formal linkage of exposure and health outcome data; indeed, there are problems inherent in surveillance of environmentally related diseases when based on formal linkage of routinely collected data.

## Established Surveillance Systems—History, Goals, and Properties

Surveillance for various specific diseases and toxic agents has become an established feature of public health systems in developed countries. The systems include sophisticated registries and monitoring networks that collect data through several different techniques.

The oldest systems that allowed monitoring of population health trends are vital statistic records established in Europe in the 1700s. In England and Wales, death records had a prominent place as demographic barometers for the health of communities and citizens throughout the 19th century. Variations in mortality rates from diseases such as cholera, dysentery, or workplace-related death (e.g., due to mining accidents) suggested socioeconomic, work-related, and environmental causes. This information was employed to justify a public health campaign not only to improve population health in England but also to measure the success of interventions [e.g., the construction of sewer systems (Mooney 1997)]. A distinguishing property of these early surveillance systems was a focus on acute causes of death for which there were either close temporal and/or spatial proximity between a perceived exposure and the outcome sufficient to establish causality (Koch, in press); or they allowed broad ecological comparisons of mortality rates between communities before and after public health interventions were implemented.

Vital statistics data (deaths, birth numbers, and outcomes) still provide a major source of surveillance data to monitor and to compare general trends in population health, to identify subgroups at risk, and to assess the effectiveness of intervention and treatment programs. Moreover, developed nations have invested in the establishment of many registry systems to collect more detailed morbidity data that provide surveillance for acute and chronic infectious diseases, occupational injuries and deaths, cancers, and birth defects. Unfortunately, in the United States none of the health outcomes surveillance databases are linked specifically, in either space or time, with relevant exposure databases.

Below we describe the function, motivation, and attributes that make these systems successful.

### Surveillance for infectious and other acute diseases.

Infectious disease surveillance is the paradigm for the surveillance of diseases that, aside from some exceptions (e.g., syphilis, tuberculosis, and AIDS), are characterized by acute onset and rapid resolution. The descriptive data provided by surveillance systems for these types of diseases provide the basis for the monitoring of the effects of interventions (e.g., standardized treatment, public education campaigns) and temporal and spatial trends that reflect changes in population behavior and attitudes, demography, provision of health services related to sexually transmitted diseases, and loss of efficacy of standard treatment regimens. The detection of “outbreaks” of disease [e.g., resurgence of syphilis in populations of homosexual males (D’Souza et al. 2003)] is an integral part of these systems. The timely collection, organization, analysis, and dissemination of these data facilitate prompt responses by public health systems to changes in disease occurrence for which immediate intervention appears warranted. The CDC’s critical role in the control of infectious diseases depends, to a large extent, on data from these surveillance systems. For these efforts to succeed, highly specific and rapid methods for recognition and unambiguous diagnosis of diseases, coupled with effective and acceptable intervention strategies, must be available. Many acute infectious diseases fulfill these requirements. Continued technological advances can be expected to improve still further the diagnostic speed for other infectious diseases that require longer diagnostic confirmation periods, such as tuberculosis and AIDS. Intervention strategies to stop outbreaks are varied and include *a*) treatment of the infected individual, which results in both the recovery of the affected individual and the protection of susceptible individuals in the population from transmission, and *b*) if no treatment is available, quarantine of the infected individual until remission and immunization of susceptible individuals—that is, by prevention of transmission of the infectious agent and disease through the removal of susceptible individuals or carriers. Surveillance of diseases for which we routinely immunize continues for the purpose of identification of gaps in immunity in a population.

Infectious diseases of more insidious onset and/or tendency to relapse and remit over long periods of time (e.g., tuberculosis, HIV infection, malaria, *Helicobacter pylori*) pose problems for surveillance and characterize many of the chronic diseases that would be the target for environmental health tracking. Although the specific pathogens can be identified with relative ease, the diseases can present slowly over long periods, such that the connection between the primary exposure sources is lost or difficult to trace with specificity. Treatment usually is long and burdensome, and methods for primary prevention may be difficult or impossible to implement (in terms of cost, acceptability, and need for persistence). For example, to control malaria, one has to prevent transmission of the disease (vector control and behavior change) rather than control the disease after transmission has occurred. Because responses for these programs do not curb the occurrence of proximal cases, the success of these interventions will often not be apparent until after a lengthy period during which no new cases are observed. In fact, in cases where there are long delays between the implementation of an intervention and the reduction in disease incidence or morbidity, it may be difficult to quantitate precisely (or even accurately) the extent to which the intervention altered the outcome of the disease.

To complicate matters further, there are a number of infectious agents that, to date, elude our diagnostic and surveillance tools. Many viruses and bacteria cause nonspecific syndromes or symptom complexes that include most diarrheal and respiratory symptoms. The situation whereby similar syndromes are caused by many different infectious agents bears a striking similarity with the situation of environmental exposure to chemical agents because many different agents or mixtures can produce a similar syndrome. New infectious agents (and, by analogy, chemical exposures) that produce these nonspecific syndromes may elude detection for long periods or until such time as a unique syndrome has been successfully related to an agent/exposure (e.g., *Escherichia coli* O157:H7c and hemolytic uremic syndrome). Although surveillance systems to monitor entire populations for these ubiquitous disease syndromes or symptoms that generally do not result in chronic illness or death have not been a priority in the past, the importance of “syndromic surveillance” has now been recognized (CDC 2004). Only when there is a small susceptible group that suffers severe symptoms or deaths do these syndromes start to draw public attention and require a response and investment in pathogen identification and disease prevention efforts (e.g., the West Nile virus outbreak; most infected individuals show minor symptoms of respiratory illness, but some infections in the elderly cause death). In general, for infectious diseases and syndromes for which we lack diagnostic and/or immunization-based prevention tools as a society, we opt for broad-based strategies to prevent exposure and intervene on potential media (e.g., prevention of contamination of water or food by microorganisms), instead of implementation of large disease- or syndrome-based surveillance.

Acute poisoning from metals or chemicals has similar attributes to infectious diseases, such as specific and acute symptom complexes that can be identified via biological tests. In the case of lead poisoning, state and federal agencies implemented a combination of preventive measures (removing lead from paint and gasoline) and surveillance for high levels of exposure that are likely in susceptible groups (e.g., in California for young low-income children with health insurance from Medi-Cal). However, there is one fundamental difference compared with the treatment of an infectious disease: Only lead removal from the environment, not the medical treatment of an individual, will reduce the risk to others in contaminated environments. Thus, the intervention that follows the identification of a poisoning case through surveillance will have to be broader and include remedial activities that remove the sources of poisoning. In fact, as we discuss below, the primary operational goal of environmental health tracking is the “treatment” of the environment in such a manner as to reduce population risk. Although a substantial part of the effort to control infectious diseases would also fall under this rubric, it is important to recognize that the individual medical treatment and prevention aspects of infectious disease surveillance are less relevant for many of the noninfectious health outcomes that will be considered for inclusion as part of environmental health tracking (e.g., asthma, many cancers).

### Surveillance for chronic diseases.

Similar to infectious and other acute disease surveillance, surveillance for chronic diseases has been implemented largely for diseases that are dreaded because of their consequences (disability and death). We define a chronic disease/syndrome as one that can have acute or insidious onset and whose symptoms and/or physiological abnormalities persist over long periods of time (years to lifelong, but they can remit with or without recurrence, e.g., asthma).

Another criterion that applies to both types of diseases for which surveillance systems exist is that they are identifiable by clinical and/or pathological examination with a high degree of specificity; that is, measurement tools are available and cost-effective and allow for unambiguous diagnosis. However, although early disease detection and intervention might be favorable and increase survival for some chronic diseases (e.g., carcinoma of the cervix, colon cancer, breast cancer), for many, neither screening tools nor universally effective treatments are available (e.g., lung cancer, many cancers of the gastrointestinal tract). Furthermore, because many chronic diseases generally are irreversible without some intervention, treatment interventions will not remove the cause of the disease in the same way as an antibiotic may eliminate bacteria and, at the same time, prevent transmission of the infection to others. However, in contrast to the specificity of metal chelation therapy in the case of lead poisoning, treatment for a chronic disease such as asthma is likely to be effective independent of the cause of the disease; for example, inhaled steroid treatment reduces inflammation and symptoms regardless of the nature of the trigger (molds, viruses, or air pollution) causing attacks.

Cancer surveillance has been described by CDC as an essential tool to *a*) assess patterns in the occurrence of cancer and detect important trends within populations, *b*) assess the impact of cancer prevention programs, and *c*) allow the rational allocation of limited resources for cancer (CDC 2004). Some of the attributes that favor certain infectious diseases for surveillance activities clearly overlap with those of certain cancers; that is, for some cancer types, effective strategies exist for reduction of mortality from cancer, and strategies for prevention of new cases may exist that include changes in behavioral and environmental factors.

Interestingly, another stated goal of cancer surveillance is the “wise allocation of limited resources including setting priorities for allocating health resources,” which depends partly on the “availability of complete, timely, and high-quality cancer data” (CDC 2004). For those cancers for which the etiology is unclear and/or complex and/or for which satisfactory early screening tools and/or treatments are lacking, surveillance data represent an important research tool for ascertainment of disease etiology (host and environmental factors) and definition of disease natural history (progress of disease over time). For those cancers for which screening is new (e.g., recommendations for colonoscopy for colon cancer) or for which the groups that derive maximum benefit are still controversial (e.g., mammography), surveillance can provide important data on the effectiveness of screening. However, if there are long lag periods between the introduction of a screening procedure and improved survival for a specific cancer, it may be difficult to quantitate the population benefit. If a new environmental exposure or some human behavior intervenes during this time interval and changes the incidence and/or the natural history of a given cancer or group of cancers, the efficacy of a screening program may be underestimated at best or considered nonexistent at worst. These issues clearly are relevant for any environmental health tracking system that focuses on chronic health problems. These issues are summarized in Table 1.

The same caveat for new screening tools also applies to the evaluation of preventive interventions. Because of the long latency between initiation and diagnosis of most cancers, the effectiveness of preventive interventions, such as the success of smoking cessation programs, will not become apparent until years or even decades after implementation. Thus, intervention evaluation efforts must operate on a different time scale from those for many acute infectious diseases. Furthermore, cancers, like most chronic diseases, have multi-factorial etiologies such that several risk factors operate through different or similar pathways that lead to the same outcome: For example, lung cancer can be caused by smoking and by exposure to asbestos, and subjects exposed to both agents may differ in risk from those exposed to either one of these carcinogens (Liddell 2001). However, surveillance of cancer trends over time that aims to document the success of an intervention could be misleading if the reduction of one of the carcinogens in a population (e.g., the prevalence of asbestos exposure) is accompanied by an increase in prevalence in another risk factor for the disease (e.g., smoking). Whether or not these two exposures would affect the same individuals in a population would not matter, because we are only monitoring trends in overall population rates.

Although cancer surveillance through registries enables a vast amount of etiologic research that contributes to the identification of cancer risk and preventive factors, this research is not part of the monitoring/surveillance effort per se but requires separately funded scientific studies, some of which will make use of surveillance data as a primary or major resource. These studies are necessary to identify the cancer-initiating events that generally precede disease diagnosis by years or decades and to estimate individual level exposures and take latency and susceptibility into account. Etiologic factors that contribute to cancers are not identifiable through disease surveillance except in those rare cases where a carcinogenic agent can be identified by a biological or chemical marker in the affected tissue(s) long after the initiation of cancer. One example is the human papilloma virus, which can be identified at higher rates in the tissue of women diagnosed with cervical cancer than among nonaffected controls (Salmeron et al. 2003). However in such cases, to permit causal inferences, a registry also would need to obtain samples from unaffected population controls, a task outside the scope of any registry. This reasoning extends also to cancer cluster investigations; that is, only when an etiology is already established and highly specific (e.g., for vinyl chloride and angiosarcoma or asbestos and mesothelioma, but not asbestos and lung cancer) can a cluster suggest the cause of the disease and be used to help guide intervention and prevention efforts (removal of asbestos). Therefore, careful consideration must be given to the expenditure of resources to investigate such occurrences.

## Environmental Health Tracking

### Use of existing surveillance systems for linkage purposes.

We have listed the goals and requirements for an EPHT system in Table 2. Generally, such a system can take advantage of already existing, active, passive, or sentinel surveillance systems, if the requirements for linkage are fulfilled (see “System requirements,” Table 2) or if they can be used as a starting point from which additional data that pertain to environmental exposures or the diseases of interest can be obtained. These systems have different functions, costs, and utility for public health and environmental tracking. Active surveillance systems have the advantage of relatively complete ascertainment and standardized collection of data over time but are very resource intensive and usually focused only on one type of disease or exposure. Passive systems are cheaper to maintain but are potentially subject to biased, incomplete reporting. Reports of unusual events (e.g., space–time clusters of disease, uncommon exposures such as a toxic spill) do not meet the formal requirements for surveillance noted above. However, the systems through which these reports appear (e.g., *Morbidity and Mortality Weekly Report*) do provide the temporal continuity and standardization of presentation that satisfy the requirements for surveillance. Reports of unusual events may provide the initial stimulus for the identification of important ongoing, environmental health risks but should avoid the pitfalls of chronic disease (cancer) cluster investigations. Most important, all existing surveillance systems, once they fulfill the requirements for linkage, can serve descriptive functions and allow the conduct of ecologic analyses in the broadest sense—that is, population exposures and outcomes for population inference. On the other hand, etiologic questions may be answerable only if additional resources become available to *a*) provide for collection of additional data for assessment of exposure and other disease risk factors (e.g., those that act as confounders or effect modifiers) at the individual level (e.g., pesticide or air pollution exposures at homes and workplaces of subjects of interest, smoking and diet information, genetic susceptibility factors, access to health care); *b*) collect data in control subjects (e.g., nondiseased subjects as controls for cancers) or collect data for diseases for which routine monitoring systems are not in place (e.g., asthma); and *c*) conduct additional data analysis not provided for within the routine monitoring systems. Furthermore, certain etiologic questions may be answered best through other types of study design that do not rely on disease monitoring in a geographically based population but either follow cohorts of individuals over a long time [e.g., Nurses’ Health Study (Hunter et al. 1990; Laden et al. 2001) cohort] and/or store biological samples for a large number of individuals [e.g., the National Health and Nutrition Examination Survey (NHANES) (Chapman et al. 2003)], or target special highly exposed groups within a population [e.g., the Agricultural Health Study for pesticide exposures (Alavanja et al. 2003)] or vulnerable subgroups of a population (children for asthma, elderly for Alzheimer or Parkinson disease). Table 3 lists the advantages and disadvantages of different systems to evaluate environmental health questions and references examples from the literature for the use of such systems.

### Criteria for the expansion/contractions of an existing surveillance system.

The design of an EPHT and surveillance system cannot be static. There always will be a need to expand the “core” of the system or to provide ad hoc elements to address specific issues whether these relate to what data the system collect or which populations it needs to cover. The criteria for expansion (and contraction) cannot be specified *a priori*; however, what can be specified is a process to keep the system dynamic and relevant. Table 4 summarizes some suggested questions that should be addressed. Most important are recognition of the need for continued re-evaluation and the existence of a base of fiscal resources to make adjustments when such are deemed necessary.

A parallel issue relates to the ability of a tracking system to recognize the potential for some new environmental exposure to cause health effects before adequate human health data are available. The solution to this problem is the inclusion of a mechanism for ongoing, continuing reviews of the relevant toxicology and exposure literature. The regular preparation of position papers by expert panels should serve as the first step in the decision-making process that is identified by item 1 in Table 4. The findings of these position papers should be subjected to a second-level review to assess the logistical and cost–benefit implications of the inclusion of new exposures into the tracking system.

## Conclusions and Recommendations

Initiation of linkage between existing disease and exposure surveillance systems for EPHT is very desirable and feasible. We have identified what we believe to be the important pitfalls that should be avoided for such linkage activities. The goals, purposes, and limitations of any proposed linkage must be discussed and stated clearly. In addition, currently available data resources and surveillance systems will need to be evaluated critically first to decide whether they fit the criteria for a successful linkage or might need to be updated and expanded to make linkage possible and useful.

Identification of many important relations between environmental factors and heath outcomes requires individual-level data that are not routinely collected by any surveillance system; thus, these can be addressed adequately only with targeted research. In contrast, data linkages performed in a surveillance context typically will not be able to address key factors at the level of the individual. Data linkage efforts may be able to detect some relations but would also be expected to miss others that could, however, be established in well-designed epidemiological studies. The distinction between data linkages in the surveillance context and targeted research is an important one, and the EPHT program must avoid the expectation that simple linkage approaches in the surveillance context can substitute for sound epidemiological research.

Design of surveillance approaches requires a balance between demands for more extensive and higher quality data and the feasibility of collecting such data. For environmental agent–disease relationships that are already well established, formal linkage of data may not be the most efficient use of resources. For example, exposure to lead has been clearly associated with decreased cognitive development in children. Use of data linkage projects to assess this relationship at the community level might be problematic because of our potential inability to detect subtle but important effects that require large cohorts of children and very sophisticated test procedures, and resources might be better devoted to identifying and addressing determinants of exposure. Furthermore, tracking of exposures to environmental agents without linkage to health outcomes can spawn effective interventions, such as efforts to reduce the use of polybrominated diphenyl ethers after these compounds were detected in increasing concentration over time in human breast milk.

Concerns about the implications of data linkage are particularly important in a policy context. A community-level association between exposure to an environmental hazard and an adverse health outcome need not be demonstrated before intervention is initiated if the relationship has been appropriately established in the scientific literature. For example, not every community needs to show a relationship between consumption of local fish contaminated with mercury and elevated blood mercury levels before taking action to warn the population that excessive local fish consumption should be avoided. Moreover, as we discussed above, for chronic diseases of multifactorial etiology, it will be difficult to demonstrate relationships between reductions in releases or concentrations of environmental agents and disease outcomes. The U.S. EPA is beginning to emphasize “accountability”—that is, demonstrations that reductions in health outcomes result from policy activities that reduce levels of hazardous agents in the environment. Although it is laudable to show such relationships where they can be demonstrated, the converse view that such relationships must be demonstrated before a policy intervention can be initiated is not supported.

Because chronic, multifactorial diseases do not lend themselves to data-driven, quick, and convenient evaluations of intervention strategies, time trends, exposure identification, or the identification of at-risk populations based on linkage and surveillance only, we propose that, first and foremost, EPHT should be synonymous with a dynamic process that requires regular system updates to *a*) incorporate new technologies to improve exposure and disease assessment at the population level, *b*) allow public dissemination of new data that become available, *c*) allow the public health and environmental policy communities to address new and emerging “threads” (for both exposures and health outcomes), and *d*) evaluate its effectiveness over some appropriate time interval. A challenge will be to maintain consistent support and funding for important routine public health systems that may seem less exciting than the public outrage producing “toxins or diseases of the week.” This is particularly true at times of economic downturns, in response to short-term public and political pressures. Although the risks attributable to environmental factors might be small in a relative sense, they can result in a large disease burden in absolute numbers because of the ubiquitous nature of certain exposures, the possible synergy of these factors with other risk factors, and the increased vulnerability of certain subpopulations. Thus, risk assessments based on any single surveillance system are likely to provide downwardly biased estimates of risk for a specific environmental hazard, because of the difficulty related to the identification of the effects of exposures to multiple environmental hazards whose composition may change over time and for which it is nearly impossible to construct accurate exposure histories even at an ecological level. Nonetheless, in some cases, surveillance may be the only practical method to obtain sufficient data to carry out a preliminary assessment of risk (contingent on adequate quality data).

By their nature, many chronic diseases are irreversible to a large extent (if at all) even after the exposure is removed. Therefore, treatment interventions directed at individuals will not remove the cause of the disease or the possible source of disease for others in the community. Thus, the primary operational goal of environmental health tracking has to be the “treatment” of the environment in such a manner as to reduce population risk. It will be important and necessary to evaluate and weigh the benefits of surveillance against its costs. In addition, we have pointed out that some strategies to evaluate the effectiveness of interventions can be severely flawed if they do not address the complexity of disease causation. On the other hand, prevention might be our only rationale option, even if multifactorial diseases do not lend themselves to surveillance data-driven evaluations of intervention strategies.

## Figures and Tables

**Table 1:** Challenges for chronic disease surveillance relevant to EPHT.

Characteristics of the disease
Onset can be insidious
Exact time of onset not known and often not subject to estimation, which complicate temporal characteristics of exposure
Often long latency between onset of exposure and clinical manifestation of disease
Heterogeneous mix of phenotypic components (e.g., asthma: allergic, nonallergic, cough variant types)
May have multiple natural histories and differ in antecedent exposure profiles (risk factors for onset or recurrence)
Genetic heterogeneity may not be reflected in phenotype (e.g., young-onset breast cancers with and without *BRCA1* and *BRCA2* mutations)
Multiple etiologies; some pathways may not involve the same putative risk factors (e.g., young-onset Parkinson disease caused by MPTP exposure or by Parkin mutations)
Characteristics of exposure
Often involves complex mixtures that can change over time
Relevant parameters often not easily defined
Timing of onset
Cumulative dose versus critical time of exposure
Threshold versus no threshold
Effect modification by other exposures
Direct measurement often not available
Reliance on imperfect surrogates

MPTP, 1-methyl-4-phenyl-1,2,3,6-tetrahydropyridine.

**Table 2:** Goals and requirements for an EPHT system.

Surveillance goals	Requirements for health data	Requirements for exposure data	System requirements
Descriptive (ecologic)	Chronic diseases	Long-term exposure assessment	Minimum set of variables for linkage is available (e.g., residential/work address and geocoded exposure location)
Temporal	Specificity of diagnosis	Broad spatial coverage that captures medium-scale spatial heterogeneity and should “match” health spatial units as closely as possible	Clear documentation of all variable of changes in format and/or content
Spatial	Standardization of diagnostic algorithms over time and procedures to convert from one standard to another (e.g., ICD-9 to ICD-10)	Long historical record keeping and acceptable procedures to convert old to new measurement techniques or metrics	Continued linkage of health and exposure data
	Moderately short time delays between diagnosis and “registration” (e.g., example within 6 months)	Develop criteria for selection of exposures such as known or suspected health impacts and/or regulatory requirements	Continued dissemination of results to agencies and public
	Agreed upon spatial reference (e.g., residence at diagnosis)	Identification of “sentinel” substances where possible	Ongoing administrative, legal, and fiscal support for linkage and dissemination activities
	Acute diseases (e.g., poisonings)	Collect data on	
	Specificity of diagnosis	Broad categories of sources	
	Standardization of diagnostic algorithms over time and procedures to convert from one standard to another (e.g., ICD-9 to ICD-10)	Broad classes of relevant “components”	
	Short time delay between identification and registration (e.g., days to weeks)		
	Agreed upon spatial reference (e.g., residence at diagnosis)		
Etiologic	Chronic and acute diseases and clusters	Requirements in addition to those mentioned above	Requirements in addition to those mentioned above
Chronic	Specificity and standardization (as needed for descriptive purposes)	Near real-time or real-time access to quality-assured data for acute disease and cluster evaluation	Ability to acquire QA/QC and release data consistent with time requirements
Acute	Time of registration and spatial reference (as above)	Ability to estimate individual exposure for acute, cluster and chronic disease, or refined spatial and temporal data for acute disease and cluster evaluation	Ability to support special monitoring projects
Clusters (spatial and temporal)	Expanded data on risk factors	Data sufficient for spatial and temporal (acute and cumulative) exposure modeling over time for chronic disease	Fiscal and staff support for ongoing modeling
	Access to noncases for risk factors and exposure	Specific source apportionment in terms of sources and components for acute disease and cluster evaluation	Fiscal support for selected, existing registries and special studies

Abbreviations: ICD, *International Classification of Diseases, 9th and 10th Revisions* (WHO 1978, 1993); QA/QC, quality assurance/quality control.

**Table 3:** Advantages and disadvantages of various systems for the examination of environmental health questions.

Registries	Advantages	Disadvantages	Selected examples/references
Disease registries			
Death or birth certificates	Standardized continuous collection of data for the total population in a geographic area Collects causes of deaths, birth weight, and gestational age in a standardized manner Allows examination of differences in space and time that includes trends for causes of deaths and birth outcomes Relatively cheap and well established	Outcome data are relatively limited in breadth (i.e., to fatal diseases and few birth outcomes) Relatively little quality control over data collection No exposure data Automatic link to exposure data possible through address (at birth or deaths) For extensive individual level exposure assessment, subjects (or proxies) need to be contacted (additional research funding necessary) Potential ethical and legal concerns related to automatic data linkage	Ritz et al. 2000 Wilhelm and Ritz 2003
Disease registries (reportable infectious diseases, cancer, end-stage renal disease, and birth defect registries; hospital discharge data; health maintenance organization data)	Standardized continuous collection of data for the total or subgroups of a population in a geographic area Allows examination of differences in space and time that includes trends for these diseases High-data quality for registries established in accordance with specified (national) standards (e.g., surveillance epidemiology and end results cancer registry standards)	Laws necessary that mandate reporting and registration Continuous and extensive financial support necessary Often registers only one specific type of disease No exposure data available Automatic link to exposure data possible through address at diagnosis (additional research funding necessary) Potential ethical and legal concerns related to automatic data linkage	Ritz et al. 2002 Reynolds et al. 2003 Shaw et al. 1999 Mann et al. 2002
Exposure/hazard registries			
Ecological exposure registries/databases (air and water pollution, pesticides, industrial emissions inventories)	Standardized continuous collection of exposure data for the total population in a geographic area Allows examination of differences in space and time that includes trends for these exposures High-data quality for these registries based upon certain specified (national) standards Allows for population-level exposure estimates either directly or through model	Laws necessary that mandate reporting and registration Continuous and extensive financial support necessary Usually registers only one specific type or group of exposures in a single medium (e.g., air, water) Exposure data are collected at the ecological not at the individual level No disease information without additional linkage to geographic identifiers (e.g., addresses) For disease outcome, linkage subjects (or proxies) may need to be contacted (additional research funding necessary)	Ritz and Yu 1999 Mortimer et al. 2002
Individual-level exposure registries (biomonitoring, e.g., NHANES)	Collects specific exposure data for a group of select individuals suspected to be exposed at high levels, or for a regional or national random sample of the population Allows examination of exposure differences in space and time that includes trends for exposures if collected repeatedly or continuously Individual-level exposure measurements available Specific exposures of relatively high data quality	Very expensive Usually only one type of specific exposure collected Usually no disease data collected simultaneously or prospectively (needs addition research funding) If samples are collected for specific research purposes only, subjects need to consent to new analyses Groups that are willing to contribute urine, blood, etc., may not be representative of the larger population	Murphy et al. 1983 MacIntosh et al. 1996 Ruckart et al. 2004
Surveys			
Cross-sectional or repeated surveys (NHANES, ISAAC, MONICA, CHIS)	Collect data on one or more diseases and exposures simultaneously for a representative regional, national, or international sample using standardized methods Allows examination of differences in space and time including trends for exposures and diseases if collected repeatedly Individual-level exposure and disease measures available High-data quality Subjects need to be contacted and participate only once	One time or repeated high financial investment necessary; costs depend on data collection protocol, sample size, length of observation period, etc. Cross-sectional data for exposure and disease may cause problems of temporal ambiguity and survivor bias Disease outcome measures often rely on self-report only Research subjects have to be willing to participate, thus may not be representative of the general population	Keil et al. 1996 Hirsch et al. 1999 Ramadour et al. 2000 Peters et al. 2001 Chapman et al. 2003
Longitudinal cohorts (Framingham study, Nurses’ Health Study, California Teachers Study, Agricultural Health Study)	Collect one or more diseases and exposures over time Longitudinal data for exposure and disease avoid problems of temporal ambiguity Investigation of outcomes beyond those of original interest often can be undertaken Individual-level exposure estimates available High-data quality	Extremely high financial investment necessary over extended periods; costs depend on data collection protocol, sample size, length of observation period, etc. A cohort is by definition a restricted group of individuals that may or may not be representative of a population of specific interest (e.g., those highly exposed to an environmental agent or those within a susceptible age or ethnicity) Research subjects have to be willing to participate repeatedly over extended periods of time and have to be traceable The study protocol dictates exactly for which disease and exposures information will be collected, unless biological samples can be stored for later analyses (which may have legal implications for consent)	Hunter et al. 1990 Garland et al. 1995 Feskanich et al. 1998 Laden et al. 1999, 2001 Alavanja et al. 2003

Abbreviations: CHIS, California Health Interview Survey; ISAAC, International Study of Asthma and Allergies in Childhood; MONICA, Monitoring of Trends and Determinants in Cardiovascular Diseases.

**Table 4:** Issues for expansion and contraction of an EPHT system.

Have scientific data provided compelling new evidence of disease–exposure associations or evidence that previously suspected associations are not causal? Are there new technologies (biomarkers, molecular dosimeters) that indicate the need to updated data collection procedures? Have there been changes in nosology that require new case definitions? Are there new sources of ongoing data collection (routine public health, research cohorts) that offer cost-efficient opportunities to expand surveillance activities? Have there been changes to sources of exposure data that either improve their quality or render them no longer suitable for routine surveillance? Is there public concern about an environmental health issue for which surveillance is the most efficient mechanism to acquire preliminary data? Is there widespread use of a new substance/chemical with the potential for exposing a large population or biopersistence of a substance (e.g., phthalates)?
